# A cell-based model approach to radiation-induced breast cancer development

**DOI:** 10.3389/fpubh.2026.1802056

**Published:** 2026-05-11

**Authors:** Jins de Jong, Jesse Vogel, Andreas Spörl, Nikolas Pomplun, Christine E. Hellweg

**Affiliations:** 1 Netherlands Organisation for Applied Scientific Research (TNO), Amsterdam, Netherlands; 2 German Aerospace Center (DLR), Space Operations and Astronaut Training, Oberpfaffenhofen, Germany; 3 German Aerospace Center (DLR), Institute of Aerospace Medicine, Cologne, Germany

**Keywords:** breast cancer, carcinogenesis, cell model, numerical approximation, radiation exposure, simulation method

## Abstract

Epidemiological studies, e.g., on atomic bomb survivors, have shown that exposure to ionizing radiation increases the risk of developing breast cancer. Typically, these studies correlate intrinsic and extrinsic contributing factors with the observed cases of breast cancer in a defined population. In this study, we attempt to model these relationships at the cellular level. For this, a nine-stage model of the epithelial cells in the milk ducts is developed, considering transition probabilities between the stages. As a proof of principle, the transition probabilities between the stages of our model are fit for scenarios with and without exposure to ionizing radiation. Although this model simplifies the complex interactions of external and internal factors and diverse cell types, we believe that these methods can be used to understand carcinogenesis from a different perspective.

## 1 Introduction

It is well-known that exposure to ionizing radiation increases the probability of developing cancer (1, 2). It is essential to understand and control these risks for people who have been exposed to ionizing radiation at levels above the natural background on Earth. This is the motivation for this study. Instead of using phenomenological relations between the estimated or measured dose received and the number of observed cases, we attempt to simulate cellular responses and outcomes during and after radiation exposure. Since cancerous cells may only appear years after radiation damage has been inflicted, we investigate a scenario of temporarily increased radiation exposure, such as those resulting from medical treatments, after nuclear reactor accidents, or during space travel.

Typically, such simulations are performed using Monte Carlo simulations. Although these are very versatile and effective, the computational power required for large-scale simulations may quickly become problematic. Therefore, we apply an approximation method that is able to estimate the probability of developing cancer much faster. In addition to this approximation, we exploit the symmetries of the model to reduce the computational effort even further.

**Figure 1:**
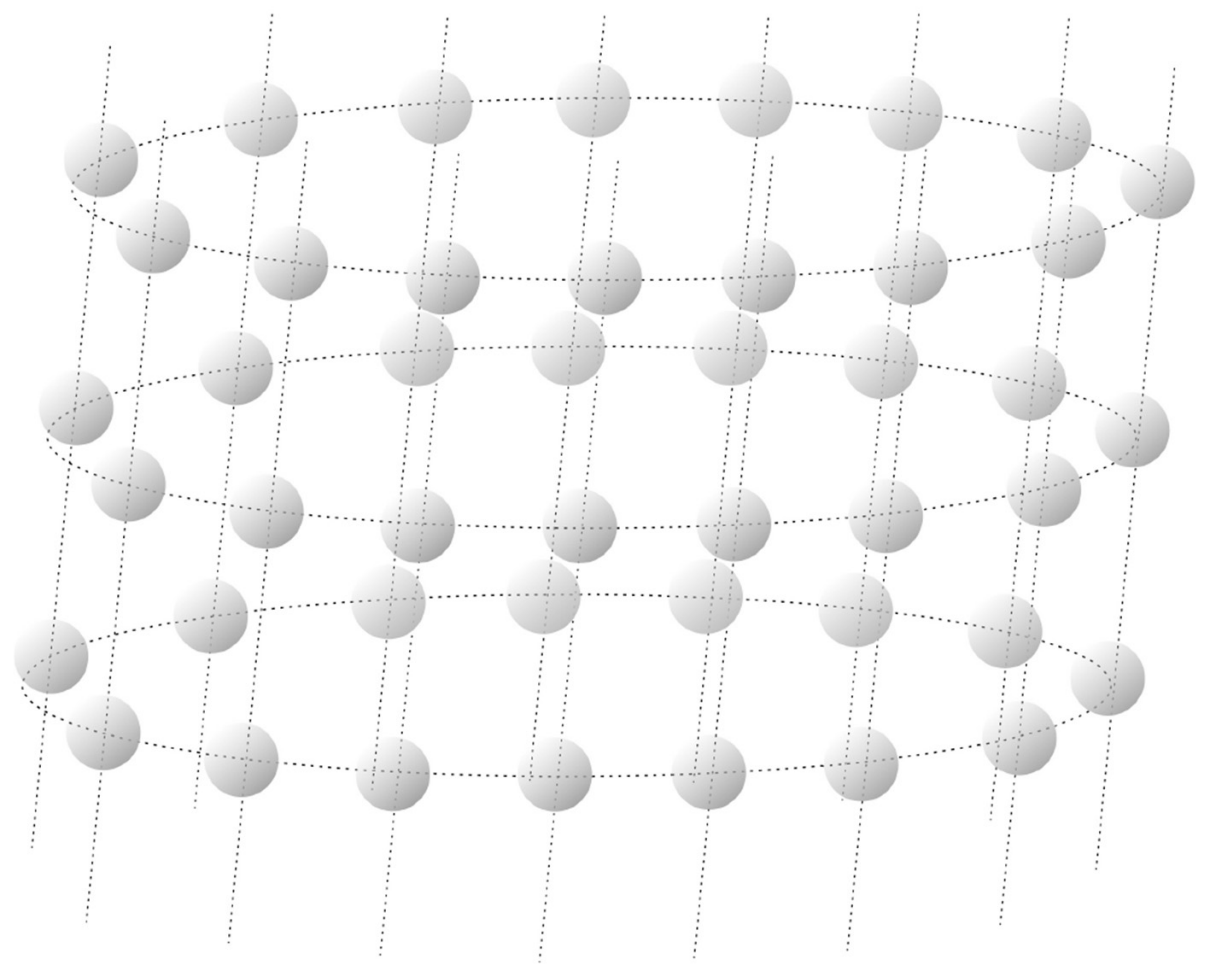
Three layers of the lattice used to model the ducts. The dashed lines indicate neighboring cells. The gray spheres indicate the cell nuclei.

The use case for this project is breast cancer originating from the milk ducts in the breast, i.e., ductal carcinoma. This develops predominantly in the single layer of epithelial cells forming the inner side of the milk duct. We approximate these layers as long tubes of aligned cells, as shown in Figure 1. This provides our use case with rotational and translational symmetry, meaning that all cells have the same probability of being in a certain state. As a result, only a single cell needs to be considered in our simulations, which results in a considerable speed-up.

The use case considered as an example of our method is radiation-induced cell damage. It is well-known that space travel exposes astronauts to considerable radiation doses, leading to an increased risk of developing radiation diseases, such as cancer. To determine the health risks for the astronauts caused by space radiation exposure, top-down models, such as the NASA Space Radiation Cancer Risk model (3), are used. Top-down, in this context, means that the risks are derived from epidemiological studies and transferred from one population (Japanese atomic bomb survivors) to another population (astronauts) considering differences in the exposure characteristics (radiation quality, dose rate, total dose, dose distribution in the body) and between the populations (genetic, epigenetic and environmental factors), while the cellular and molecular level is not considered directly. They are based on assessing the radiation exposure during a specific mission by radiation transport models, and assigning a health risk, e.g., cancer risk from epidemiological data (Japanese atomic bomb survivors), which are translated to the specific radiation quality in space by means of a Quality factor. This Quality factor is derived from a large set of radiobiological experiments with space-relevant radiation qualities such as heavy ions of different energies (4–6). Furthermore, a dose and dose rate reduction factor is applied to account for the lower dose rates of exposure during space missions compared to atomic bomb explosions.

For a better understanding of the risks, alternative methods, such as a bottom-up approach for specific organs, could be used. Bottom-up means in this context from the individual cell that was hit by an energetic particle to the whole organ and finally the whole organism, e.g., the astronaut. Such models implement insight into cancer initiation and promotion by space radiation (7) and may improve risk assessment by considering available knowledge on early and late cellular reactions to space radiation exposure. However, developing such models requires large-scale simulations, which are expensive and time-consuming with standard methods. This is the main motivation to study faster methods.

The scenario considered looks as follows. As the risk of malignant transformation varies between organs, it has to be determined for each organ or organ system. Here, the approach is exemplified for the breast. Cancer of the breast arises from breast epithelial cells that acquire genetic alterations in tumor suppressor genes or oncogenes (8, 9). The breast of an astronaut is exposed to a certain dose of radiation during a space mission. The genetic changes this induces will not be visible directly, but may lead to cancer development in the future. In general, breast cancer risk is associated with age, menopausal status, family history of breast cancer (10), and lifestyle factors. A small percentage of breast cancer patients have a strong genetic predisposition [mutations of the BRCA1 and BRCA2 genes, or of the ATM (11, 12) or TP53 gene (13)]. Breast cancer is most often sporadic with mutations in other genes than BRCA, and predispositions might be caused by mutations in other DNA repair genes (14), or in genes that control the cell cycle (15).

To test our approximation method, a radiation-induced cell damage model will be used. This model will be presented in Section 2.1. It differs in various ways from reality, but this is not problematic, since the main purpose of this model is to highlight the qualities and shortcomings of our approximation method, introduced in Sections 2.2–2.4. The data and set-up of the experiment for this study are introduced in Section 3.1, and the results are presented in Section 3.2. This study is concluded in Section 4.

The contribution of this study is that we demonstrate that an efficient evaluation of larger models is possible with numerical approximation techniques. Instead of the Monte Carlo simulation of a model, we consider a simulation of the individual cells. This is done in a highly symmetric case that allows us to massively reduce the computation time needed. Furthermore, our model may be seen as a quantified version of the phenomenological two-stage clonal expansion model^1^ (17, 18). The data and model used in this study are too limited to draw biologically relevant conclusions from these experiments.

## 2 Method

### 2.1 The cell model

To test the approximation method, a model for cells and radiation damage is used. This model is simplified in several aspects. The milk duct is considered a cylinder with 15 cells forming a single ring. The inner surface of the milk duct is covered by mammary epithelial cells with three major cell types: mammary stem cells-enriched basal cells, luminal progenitors (LPs), and mature luminal cells that form the inner layers of cells that line the ducts (19, 20). The basal stem cells generate myoepithelial cells that surround the luminal cells and can contract, and LPs. Mature luminal cells in ducts of the resting adult gland (i.e., one that is not currently in a gestation, lactation, or involution state) and mature lactocytes during lactation derive from LPs (19, 21). For modeling convenience, and as it is not fully known which normal cell in the breast is the key target for malignant transformations^2^ (9), we do not differentiate between these cell types and between the different maturation stages of the breast (after birth, pregnancy, lactation, involution, different stages of the ovulatory cycle, pre-, peri-, and post-menopause) and assume that the cells lie in a regular cubical lattice, as depicted in Figure 1. Approximating the cylinder as infinitely long results in translational and rotational symmetry. This makes each cell identical, which simplifies the computations significantly.

**Figure 2:**
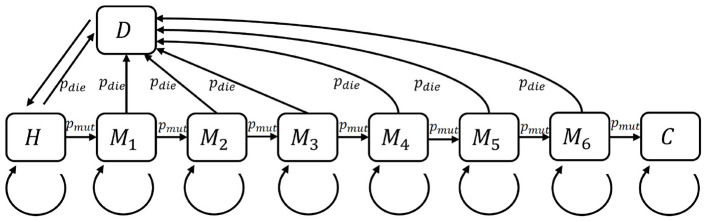
The states of a single cell as mentioned in Table 1 with their transition probabilities. Cancerous cells that were not affected by the immune system are considered the final state. *H*, healthy; *M*, mutated; *C*, cancerous; *D*, dead.

In this simplified model, cells can be in nine different states as depicted in Figure 2. The default state is the healthy (*H*) state. Spontaneously or through radiation-induced processes, the cell may acquire growth-promoting mutations. Accumulation of such mutations might lead to the loss of growth control, a hallmark of cancer cells. In this model, a cell that has undergone seven relevant mutations is considered cancerous. This means that there are six mutated cell states (*M*_1_-*M*_6_) and the cancerous state (*C*). As a result of a mutation that does not promote malignant transformation, a cell may induce programmed cell death or may be recognized by the immune system as hostile. These cells will die, e.g., by apoptosis or mitotic catastrophe, which is the 9^th^ state (*D*) in this model. Dead cells will be removed by the immune system and, depending on the regenerative potential of a tissue, replaced by a new healthy cell.

The following transitions should be recognized. There is a transition probability *p*_mut_ that a growth-promoting mutation occurs, e.g., in the proto-oncogene c-MYC (2), and the result is stable and long-living. Based on available epidemiological data on breast cancer incidence after exposure to ionizing radiation in the course of medical treatment, after nuclear reactor accidents, or by the Japanese atomic bombings, we know that *p*_mut_ is not only dose- and radiation quality-dependent, but also age-dependent (24–29). The radiation quality, based on linear energy transfer (LET), affects the spatial distribution of ionizations. Dense ionization tracks caused by high-LET radiation are a cause of more clustered (complex) DNA damage that is difficult to repair and can lead to genomic instability (30, 31). The transition probability *p*_mut_ might also be influenced by the availability of DNA double-strand break repair pathways: Non-homologous end joining is error-prone and available throughout the cell cycle, while homologous recombination can be error-free but is only available in mammalian cells when the sister chromatid is synthesized, meaning during S, G2 phase, and mitosis (32, 33). Genetic defects in these pathways, such as BRCA1/2 deficiency, result in impaired homologous recombination (34, 35), meaning that the cells have to rely on error-prone non-homologous end joining and alternative end joining. In this model, an average probability of growth-promoting mutations induced by radiation exposure was assumed for all cell cycle stages and genetic states.

**Table 1:** Left: Intrinsic transition probabilities ℙ(*E*_*t*_∣*E*_*t*−1_) between the states of cell *i* at timestep *t* (rows) and timestep *t*−1 (columns). *s* = 1−(*p*mut+*p*die), all empty fields are zero. Right: Spreading transition rates between the states of cell i at timestep *t* (rows) and cell j at timestep *t*−1 (columns) that contribute to ℙ(*E*_*t*_∣*E*_*B, t*−1_). All empty fields are zero.

*x*_*i, t*−1_ *x*_*i, t*_	H	M_1_	M_2_	M_3_	M_4_	M_5_	M_6_	C	D		*x*_*j, t*−1_ *x*_*i, t*_	H	M_1_	M_2_	M_3_	M_4_	M_5_	M_6_	C	D
*H*	s								1		*H*									
*M*_1_	*p*mut	s									*M*_1_		*p*spr							
*M*_2_		*p*mut	s								*M*_2_			2*p*spr						
*M*_3_			*p*mut	s							*M*_3_				3*p*spr					
*M*_4_				*p*mut	s						*M*_4_					4*p*spr				
*M*_5_					*p*mut	s					*M*_5_						5*p*spr			
*M*_6_						*p*mut	s				*M*_6_							6*p*spr		
*C*							*p*mut	1			*C*									
*D*	*p*die	*p*die	*p*die	*p*die	*p*die	*p*die	*p*die		0			*D*								

**Table 2:** The possible stable states of a node in the radiation-induced cell damage model.

State	Description
*H*	Healthy
*M*_1_	1 growth-relevant Mutation
*M*_2_	2 growth-relevant Mutations
*M*_3_	3 growth-relevant Mutations
*M*_4_	4 growth-relevant Mutations
*M*_5_	5 growth-relevant Mutations
*M*_6_	6 growth-relevant Mutations
*C*	Cancerous
*D*	Dead

Alternatively, mutations may result in a cell with genomic instability or eventually cell death. The probability for the latter to occur is *p*_die_. This probability depends on extrinsic factors (radiation quality, dose rate, fractionation) and intrinsic factors. An important intrinsic factor is the cell cycle stage of the cell hit by radiation. Cell cycle stage determines sensitivity to cell killing by radiation, with the highest sensitivity during the G2 phase and mitosis, and resistance during the late S phase (36). Therefore, in the epithelial compartment of the breast, several subpopulations with differing radiosensitivity can be expected, and the percentage composition depends, among other things, on the tissue type, developmental stage, menstrual cycle, breastfeeding, and menopausal status. Additionally, radiation exposure can induce cell cycle delays (36) and thereby transiently alter the percentage composition of cell cycle stages in the tissue. Furthermore, genetic factors such as ATM heterozygosity can increase the risk of radiation-induced genomic instability (37). For simplification of the model, we consider a probability of cell killing that is averaged over all cell cycle stages and genetic states.

**Figure 3:**
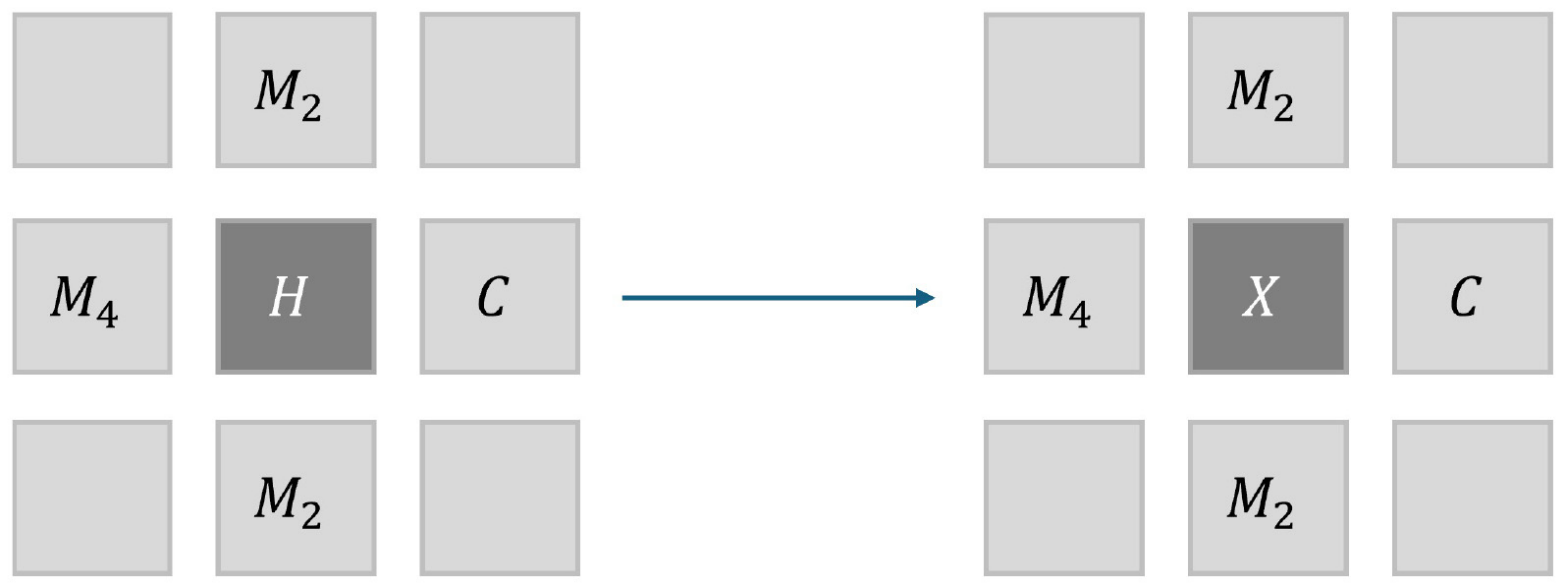
The possible states a healthy state *H* can evolve into depend not only on the cell model, but also on the state of the neighboring cells. The spreading of mutations that are relevant for cell division is modeled as spreading to neighboring cells. The probability of spreading is proportional to the number of mutations. Contrary to intuition, cancerous cells do not spread in this model, as this would not influence the outcome of a computation. Table 2 shows how this affects the transition probabilities.

As the mutations we consider are growth-promoting, cell proliferation dynamics might change. This is done via a spreading probability. A mutated cell may spread and replace a neighboring cell, as shown in Figure 3. The cell model has a fixed topology, making it difficult to implement the idea of (uncontrollable) cell division. As a compromise, rapid cell division is modeled as the spreading of mutated cells, resulting in the overgrowth of neighboring cells. If a cell is healthy or mutated and its neighbor is also mutated, there is a spreading probability *p*_spr_ that it too becomes mutated in the next time step as a result of the growth-promoting mutation of the neighbor cell. The more mutations the neighbor has, the larger the risk of spreading. For each mutated neighbor in state *M*_*n*_ for some *n* = 1, …, 6, we model this spreading probability by *n*×*p*_spr_.

Unlike all other transitions in this model, this one depends on multiple cells. The overgrowth can be explained by the faster growth of the mutated cells compared to healthy cells, leading to replacement of healthy by mutated cells during the normal tissue turnover, or even mechanical compression of the healthy cells, competition for nutrients. The cell dies, and the mutated or cancerous cell divides, and one of the daughter cells takes the place of the initial cell. Furthermore, a bystander effect might occur: the neighboring cell was hit by radiation, and damage also occurs in the cell under consideration here. The spreading probability makes the model harder to handle, so additional computational steps are needed for good results.

Both mutated and cancerous cells are defined as quite stable and long-living. However, they could be removed by the immune system if they express a tumor antigen. This means that a single cancerous cell that will escape the immune system will develop into a tumor. The question to ask during simulation, therefore, is what the probability is that at least one cancerous cell will develop and survive. The spreading probability of cancer cells correlates with the time until a tumor develops.

To implement time evolution, periodic cell renewal is considered. At the end of every time step, all cells are renewed. During a time step, the state of the cells in the duct is constant. From one time step to the next, all cell states are updated according to the transition probabilities. For these transition probabilities, there are two sets. One for normal circumstances (only natural background radiation exposure) and the other for radiation exposure above natural background at a certain dose, for example, during space travel.

As a result, the cell renewal rate remains constant during the simulation and does not become lower with increasing age. This could be corrected in a more advanced version of this model. Besides this, many simplifications are made here. These simplifications make it possible to perform fast and efficient simulations of tumor cell development in the milk duct. The purpose of this study is to see whether it is possible to gain insight into the transition probabilities at the cellular level, leading to the development of breast cancer after radiation exposure.

### 2.2 The computational model

In this section, the computational steps used to estimate the number of cancerous cells will be discussed. Before discussing the mathematical details, an overview of the method is sketched.

The computation should yield the number of breast cancer cases in an age cohort of a certain size. For this, the cells' states in the milk duct are simulated over time. When a cell becomes cancerous, this will result in a cancer diagnosis after some lag time, which we have set to 5 years. To estimate the probability that at least one cancerous cell arises in the breast tissue, we need the single-cell probability to be cancerous at a certain time in combination with the number of cells in the tissue. The symmetry of the problem allows us to perform all computations on a single cell and extrapolate the results to the entire tissue. Below, a mathematical description of the model and its evaluation is given. In this way, both the approximation and its shortcomings can be explained.

Let *N* be the number of cells that make up the tissue of interest. We denote the set of cell indices by *I* = {1, …, *N*} and the set of possible cell states by *S* = {*H, M*_1_, *M*_2_, …, *M*_6_, *C, D*}. For any simulation timestep *t* ∈ *T* = {0, 1, …, *t*_*max*_} and cell *i* ∈ *I*, let *X*_*i*_(*t*) be the *S*-valued random variable corresponding to the state of cell *i* at time *t*. In particular, the initial condition that all cells are healthy at time *t* = 0 can be expressed by the probability


$$\text{ℙ}\left(X_{i}\left(0\right)=H\right)=1\;\forall i\in I.$$

Denote by *B*_*i*_⊂*I* the set of cells neighboring cell *i*. That is, *j* ∈ *B*_*i*_ if and only if cell *i* and cell *j* are adjacent. Let *x, y* ∈ *S*^*N*^ denote the vectors describing the state of the cell system at time *t* and *t*−1, respectively. As described in Section 2.1 , the probability of finding the state *x*_*i*_ ∈ *S* of cell *i* at a time *t*>0 depends only on the state of that cell and its neighbors at time *t*−1, *y*_*i*_ and *y*_*j* ∈ _*B*__*i*__ respectively. We abbreviate the condition of *X*_*i*_(*t*) being equal to a specific state by the event *E*(*i, t*) ≡ *X*_*i*_(*t*) = *s*_*i*_. In particular,


$$\begin{array}{c} E_{t}=E\left(i,t\right)\equiv X_{i}\left(t\right)=x_{i}\\ E_{t-1}=E\left(i,t-1\right)\equiv X_{i}\left(t-1\right)=y_{i}\\ E_{B,t-1}=E\left(B_{i},t-1\right)\equiv X_{j}\left(t-1\right)=y_{j}\;\forall j\in B_{i}. \end{array}$$

The conditional probabilities


$$\begin{array}{r} \text{ℙ}\left(E_{t}|E_{t-1},E_{B,t-1}\right)\\ =\text{ℙ}\left(X_{i}\left(t\right)=x_{i}|X_{i}\left(t-1\right)=y_{i},X_{j}\left(t-1\right)=y_{j}\;\forall j\in B_{i}\right) \end{array}$$

can be expressed in terms of the intrinsic transition probabilities *p*_mut_, *p*_die_ and the spreading probabilities *p*_spr_ as described in Section 2.1 and depicted in Table 3. Summing over the neighboring cells *j* and all states *y*_*i*_ gives the probability of finding the state *x*_*i*_ of cell *i* at time *t*


$$\begin{array}{l} \text{ℙ}\left(E_{t}\right)=\underset{y_{i}\in S}{\sum }\underset{j\in B_{i}}{\sum }\text{ℙ}\left(E_{t}|E_{t-1},E_{B,t-1}\right). \end{array}$$

The following quantity is of interest to us. For any cell state *s* ∈ *S*, let *N*_*s*_(*t*) be the random variable that is the number of cells in state *s* at time *t*. For instance, due to the initial condition, we have


$$\text{ℙ}\left(N_{H}\left(0\right)=N\right)=1.$$

Clearly, the expected value of *N*_*s*_(*t*) is given by


$$\begin{array}{l} E\left(N_{s}\left(t\right)\right)=\overset{N}{\underset{i=1}{\sum }}\text{ℙ}\left(X_{i}\left(t\right)=s\right)=N\cdot \text{ℙ}\left(X_{1}\left(t\right)=s\right), \end{array}$$ (1)

where the latter equality follows from the symmetry of the model. The variance of *N*_*s*_(*t*) is given by


$$\text{Var}\left(N_{s}\left(t\right)\right)=E\left(N_{s}{\left(t\right)}^{2}\right)-E{\left(N_{s}\left(t\right)\right)}^{2}.$$

Since


$$E\left(N_{s}{\left(t\right)}^{2}\right)=\overset{N}{\underset{i=1}{\sum }}\overset{N}{\underset{j=1}{\sum }}\text{ℙ}\left(X_{i}\left(t\right)=X_{j}\left(t\right)=s\right)$$

the variance can be expressed as


$$\begin{array}{l} \text{Var}\left(N_{s}\left(t\right)\right)=\overset{N}{\underset{i=1}{\sum }}\overset{N}{\underset{j=1}{\sum }}(\text{ℙ}\left(X_{i}\left(t\right)=X_{j}\left(t\right)=s\right)\\ \;-\text{ℙ}\left(X_{i}\left(t\right)=s\right)\text{ℙ}\left(X_{j}\left(t\right)=s\right)). \end{array}$$

Due to the symmetry of the model, we may simplify this expression to


$$\begin{array}{l} \text{Var}\left(N_{s}\left(t\right)\right)=N\cdot \overset{N}{\underset{j=1}{\sum }}(\text{ℙ}\left(X_{1}\left(t\right)=X_{j}\left(t\right)=s\right)\\ \;-\text{ℙ}\left(X_{1}\left(t\right)=s\right)\text{ℙ}\left(X_{j}\left(t\right)=s\right))\\ \;=N\cdot \overset{N}{\underset{j=1}{\sum }}(\text{ℙ}\left(X_{1}\left(t\right)=X_{j}\left(t\right)=s\right)-\text{ℙ}{\left(X_{1}\left(t\right)=s\right)}^{2}). \end{array}$$ (2)

Note that for *j* = 1, we have ℙ(*X*_1_(*t*) = *X*_*j*_(*t*) = 1) = ℙ(*X*_1_(*t*) = 1).

**Table 3:** The probabilities for the state *X* in Figure 3 as a result of the transition and spreading probabilities. Normalization requires that γ = 1/(1+8·*p*_spr_).

State of *X*	Probability
*H*	γ·(1−*p*_mut_)
*M*_1_	γ·*p*_mut_
*M*_2_	4·γ·*p*_spr_
*M*_4_	4·γ·*p*_spr_
*C*	0.0

### 2.3 The single-cell approximation

In this section, we describe an approximation method that we will call the *single-cell approximation*. This approximation method is based on the following two computational assumptions.

- (*Symmetry assumption*) For all times *t* ∈ *T*, the random variables *X*_*i*_(*t*) are identically distributed for all *i*.
- (*Independence assumption*) For all times *t* ∈ *T*, we may use the following formula to compute the joint probability with *E*_*i*_ and *E*_*j*_ according to Section 2.1 :

$$\begin{array}{l} \text{ℙ}(E_{i}\wedge E_{j})=\text{ℙ}\left(E_{i}\right)\times \text{ℙ}\left(E_{j}\right) \end{array}$$

The symmetry assumption allows us to write *X*(*t*) instead of *X*_*i*_(*t*) in this section. As a consequence of the independence assumption, at any time *t*, we only need to keep track of the probabilities ℙ(*X*(*t*) = *s*) for *s* ∈ *S*.

It should be noted that the independence assumption really is an approximation, as the states of two cells are not truly independent of each other for non-zero spreading probabilities. However, we emphasize that the independence assumption does *not* mean that spreading probabilities are not taken into account at all. It is only the correlation between the states of different cells that is omitted, not the interaction between different cells. In the regime with low spreading probabilities, this approximation works quite well, but for higher spreading probabilities, the approximation starts to deviate. This will be demonstrated in Example 2 below. Indeed, this is due to the fact that the random variables *X*_*i*_(*t*) and *X*_*j*_(*t*) are not actually independent.

The state of a cell can transition from *r* ∈ *S* at time *t*−1 to *s* ∈ *S* at time *t* in one of two ways:

1. The cell has been overgrown by a neighboring cell that was in state *s* at time *t*−1.
2. The cell has not been overgrown by any neighbor, and the cell transitioned internally from state *r* to state *s*.

Writing |*B*| for the number of neighbors of any given cell, such a transition can be expressed as


$$\begin{array}{l} \text{ℙ}\left(X\left(t\right)=s\right)=\underset{r\in S}{\sum }\text{ℙ}(X\left(t-1\right)=r)\left(u_{sr}+\rho_{sr}v_{r}\right), \end{array}$$ (3)

where

- $u_{sr}=1-{\left(1-(X(t-1)=s)\tau_{sr}\right)}^{\left|B\right|}$ is the probability that a cell in state *r* is overgrown by a neighboring cell in state *s*, and
- $v_{r}={\left(\underset{q\in S}{\sum }\text{ℙ}(X\left(t-1\right)=q)\left(1-\tau_{qr}\right)\right)}^{\left|B\right|}$ is the probability that a cell in state *r* is not overgrown by any of its neighbors,
- τ_*sr*_ is the spreading probability, which is equal to *n*×*p*_spr_ when *s* = *M*_*n*_, and zero otherwise.
- ρ_*sr*_ is the internal transition probability that a cell in state *r* changes into *s*. The possible values of ρ_*sr*_ are 0, *p*_mut_, *p*_die_ or a multiple of *p*_spr_, as visualized in Figures 2, 3.

Together with the initial condition, Equation 3 defines a method to approximate the values of ℙ(*X*(*t*) = *x*) for all times *t* ∈ {0, 1, …, *T*} and all cell states *x* ∈ *S*.

Note that the value of τ_*sr*_ does not actually depend on *r*. The same holds for *u*_*sr*_ and *v*_*r*_.

In the single-cell approximation method, the expected value of *N*_*s*_(*t*) is computed from the probabilities ℙ(*X*(*t*) = *s*) as in Equation 1. The expression for the variance (Equation 2) reduces to


$$\text{Var}\left(N_{s}\left(t\right)\right)=N\cdot (\text{ℙ}\left(X\left(t\right)=s\right)-\text{ℙ}{\left(X\left(t\right)=s\right)}^{2})$$

since only the term with *j* = 1 is non-zero.

** Example 1**. Let us demonstrate the single-cell approximation method to a simplified cell mode, in which a cell is either healthy (*H*) or cancerous (*C*), that is, *S* = {*H, C*}. Healthy cells have an internal transition probability ρ_*CH*_ to become cancerous, as well as a probability τ_*CH*_ to be overgrown by a neighboring cancerous cell. We consider the following two scenarios, where it should be emphasized that the numbers are not realistic, but chosen for demonstrative purposes.

1. The case of weak interaction, where the interaction between cells, τ_*CH*_ = 0.01, is less than the internal transition probability ρ_*CH*_ = 0.05.
2. The case of strong interaction, where the interaction between cells, τ_*CH*_ = 0.05, is greater than the internal transition probability ρ_*CH*_ = 0.01.

**Figure 4:**
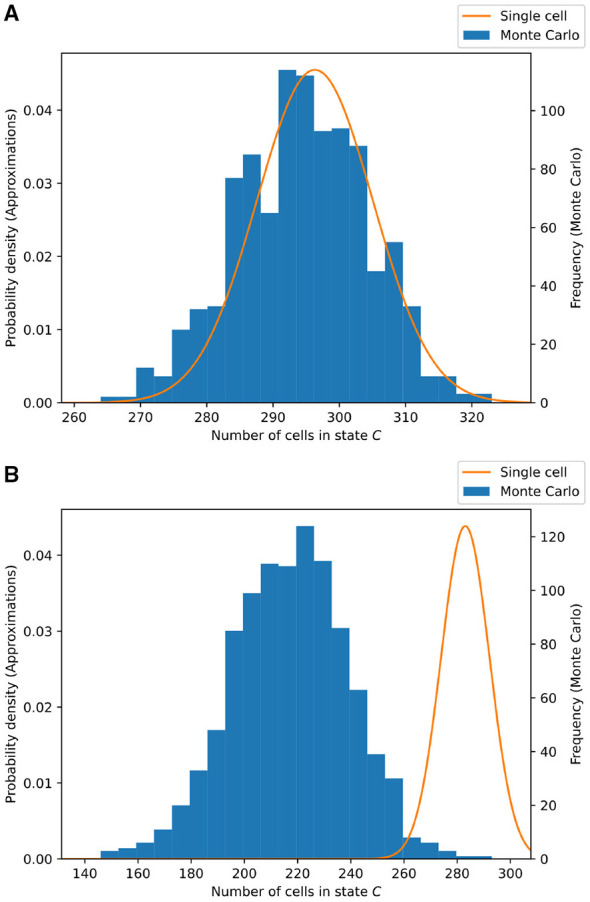
Comparison of the single-cell approximation and the Monte Carlo simulation on the two-state cell model. Two scenarios are considered: the case of **(A)** (above) “weak interaction” and that of **(B)** (below) “strong interaction.” The Monte Carlo simulation consists of 1, 000 trials of simulating 20 × 20 cells. The results are shown at time *t* = 20. The computation time of the Monte Carlo method is 75 s, and that of the single-cell approximation is 0.1 ms, on a standard office laptop. **(A)** Weak interaction: ρ_*CH*_ = 0.05 and τ_*CH*_ = 0.01. **(B)** Strong interaction: ρ_*CH*_ = 0.01 and τ_*CH*_ = 0.05. The overlap between the Monte Carlo distribution and the predicted distribution can be computed using the Bhattacharya coefficient (BC). **(A)** BC = 0.989. **(A)** BC = 0.128.

For both of these scenarios, we compare the single-cell approximation method to a Monte Carlo simulation of the cell model, the result of which can be found in Figure 4. Note that in case (*a*), the approximation works quite well, but in case (*b*), the approximation starts to deviate significantly.

In terms of computation time (with respect to a standard office laptop), the Monte Carlo simulation takes 75 s, i.e., 75 ms per trial, whereas the single-cell approximation takes 0.1 ms in total.

### 2.4 The neighboring cells approximation

To address some of the shortcomings of the single-cell approximation, we will in this section describe an improved method. This method, which we will call the *neighboring cells approximation*, is based on the following two computational assumptions.

- (*Symmetry assumption*) For all times *t* ∈ *T*, the random variables *X*_*i*_(*t*) are identically distributed for all *i*.
- (*Decoherence assumption*) For all times *t* ∈ *T*, for every sub-tissue *A*⊂*I* and every state *x* ∈ *S*^*A*^, and for every two cells *i*_1_, *i*_2_ ∈ *A* with *i*_1_≠*i*_2_ that have distance greater than 1, we may use the following formula with the definition of *E*_*A*_ according to Section 2.1 to compute the joint probability:

$$\begin{array}{c} \text{ℙ}\left(E_{A}\right)=\text{ℙ}\left(X_{i}\left(t\right)=x_{i}\text{for all}i\in A\right)=\frac{\text{ℙ}\left(E_{A\backslash \left\{i_{2}\right\}}\right)\text{ℙ}\left(E_{A\backslash \left\{i_{1}\right\}}\right)}{\text{ℙ}\left(E_{A\backslash \left\{i_{1},i_{2}\right\}}\right)}, \end{array}$$

Example 2 demonstrates this assumption.

Note that for the cells in the cylinder in Figure 1, the dashed lines indicate that two cells are neighboring. Hence, each cell has four neighbors, which are cells at distance 1, and each cell has eight cells at distance 2, and so on.

Let us elaborate a bit longer on the assumption of decoherence. This assumption tries to capture the fact that the states of cells that are further apart are less correlated. This is taken to the extreme for the single-cell approximation, as in the previous section, where different cells are assumed to be completely independent. In the neighboring cells approximation, cells are assumed to be correlated to their neighbors, but not to cells that have a distance greater than one.

** Example 2**. To make sense of the decoherence assumption as stated above, consider a sub-tissue *A* = {1, 2, 3} consisting of three consecutive cells. That is, cells 1 and 2 have distance 1, as well as cells 2 and 3, but cells 1 and 3 have distance 2. Since cells 1 and 3 have distance greater than 1, we may use the approximation


$$\begin{array}{l} \mathbb{P}((X_{1}(t),X_{2}(t),X_{3}(t))=(q,r,s))\\ =\frac{\text{ℙ}(X_{1}(t)=q\;and\;X_{2}(t)=r)\text{ℙ}(X_{2}(t)=rand\;X_{3}(t))=s)}{\text{ℙ}(X_{2}(t)=r)} \end{array}$$

for any *q, r, s* ∈ *S*.

As a consequence of the decoherence assumption, at any time *t*, we only need to keep track of the probabilities


$$\begin{array}{l} \text{ℙ}\left(X_{i}\left(t\right)=q\text{and}X_{j}\left(t\right)=r\right) \end{array}$$ (4)

for all states *q, r* ∈ *S* of any two neighboring cells *i* and *j*, and of the probabilities ℙ(*X*(*t*) = *s*), for all *s* ∈ *S*. Note that by symmetry considerations, we only need to consider the case *i* = 1 and *j* = 2.

The probability that a cell is in state *x* ∈ *S* at time *t*≥1 can be expressed completely analogous to Equation 3. The same holds for the joint probabilities (Equation 4).

Together with the initial condition, these equations approximate the values of ℙ(*X*(*t*) = *s*) and ℙ(*X*_1_(*t*) = *q*and*X*_2_(*t*) = *r*) for all times *t*≥0 and cell states *q, r, s* ∈ *S*. From these probabilities, we can compute the expected value of *N*_*s*_(*t*) as in Equation 1.

The expression for the variance (Equation 2) is not feasible to compute, as it consists of too many terms. However, the difference between ℙ(*X*_*i*_(*t*) = *X*_*j*_(*t*) = *s*) and ℙ(*X*_*i*_(*t*) = *s*)ℙ(*X*_*j*_(*t*) = *s*) tends to zero quickly as the distance between cell *i* and cell *j* increases. Therefore, a good approximation is obtained by considering only cells *j* with a distance *d* ≤ *D* from cell 1. For practical purposes, we take *D* = 4. This way, we obtain extra correction terms with respect to the single-cell approximation. In particular, we have


$$\begin{array}{l} \text{Var}\left(N_{s}\left(t\right)\right)=N\cdot (\text{ℙ}\left(X_{1}\left(t\right)=s\right)-\text{ℙ}{\left(X_{1}\left(t\right)=s\right)}^{2})\\ \;+N\cdot M_{d}\cdot \overset{D}{\underset{d=1}{\sum }}\underset{y\in S^{\left\{2,\ldots ,d\right\}}}{\sum }\text{ℙ}(X_{1}\left(t\right)=s\text{and})X_{2}\left(t\right),\ldots ,\\ X_{d}\left(t\right)(=y\text{and}X_{1+d}\left(t\right)=s) \end{array}$$

where *M*_*d*_ is the number of neighbors that a cell has at a distance *d* away. E.g., *M*_1_ = 4 and *M*_2_ = 8. Using the independence assumption, this reduces to


$$\begin{array}{l} \text{Var}\left(N_{s}\left(t\right)\right)=N\cdot (\text{ℙ}\left(X_{1}\left(t\right)=s\right)-\text{ℙ}{\left(X_{1}\left(t\right)=s\right)}^{2})\\ f+N\cdot M_{d}\cdot \overset{D}{\underset{d=1}{\sum }}\underset{y\in S^{\left\{2,\ldots ,d\right\}}}{\sum }\frac{\overset{d}{\underset{i=1}{\prod }}\text{ℙ}(X_{i}\left(t\right)=y_{i}\text{and}X_{i+1}\left(t\right)=y_{i+1})}{\overset{d}{\underset{i=2}{\prod }}\text{ℙ}(X_{i}\left(t\right)=y_{i})} \end{array}$$

where we use the notation *y*_1_ = *y*_*d*+1_ = *s*.

**Figure 5:**
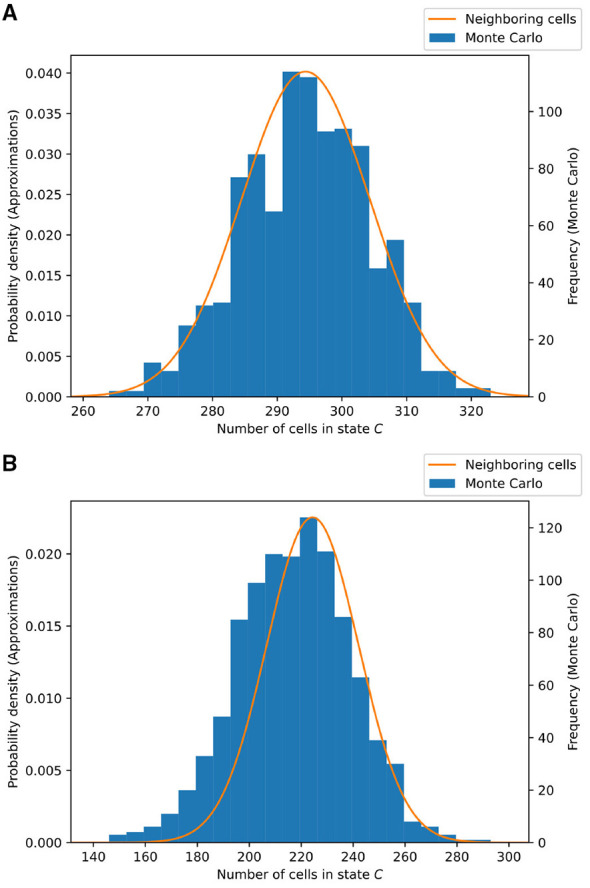
Comparison of the neighboring cells approximation and the Monte Carlo simulation on the two-state cell model. Two scenarios are considered: the case of **(A)** (above) “weak interaction” and that of **(B)** (below) “strong interaction.” The Monte Carlo simulation consists of 1, 000 trials of simulating 20 × 20 cells. The results are shown at time *t* = 20. The computation time of the Monte Carlo method is 75 s, and that of the neighboring cells approximation is 0.5 ms, on a standard office laptop. **(A)**Weak interaction: ρ_*CH*_ = 0.05 and τ_*CH*_ = 0.01. **(B)** Strong interaction: ρ_*CH*_ = 0.01 and τ_*CH*_ = 0.05. The overlap between the Monte Carlo distribution and the predicted distribution can be computed using the Bhattacharya coefficient (BC). **(A)** BC = 0.999. **(A)** BC = 0.971.

** Example 3**. Let us repeat the experiment as described in Section 2.1 , now comparing the neighboring cells approximation to the Monte Carlo simulation. All parameters are kept the same, and the results are shown in Figure 5. Compared to the single-cell approximation, as depicted in Figure 4, a significant improvement in the case of strong interaction is visible. Furthermore, this method gives a better estimate for the variance of the distribution.

In terms of computation time (with respect to a standard office laptop), the Monte Carlo simulation takes 75 s, i.e., 75 ms per trial, whereas the neighboring cells approximation takes 0.5 ms in total.

## 3 Results

The ambition of this study is to demonstrate that it is feasible to determine the transition probabilities for growth-promoting cell mutations that may result in breast cancer. There are many studies emphasizing the many factors that contribute to this. It is beyond the scope of this study to include them all. Only the influence of ionizing radiation is treated in this study.

### 3.1 Data

This immediately points to a problem. How to obtain useful data in which (ideally) only the radiation exposure is different? Large groups of women exposed to radiation can be found as a result of the Chornobyl fallout (38, 39) and the survivors of the atomic bombs in Japan (17, 40, 41). However, for a variety of reasons, it is difficult to compare groups of subjects that are spread geographically, culturally, and over decades (42). It is not the ambition of this study to tackle this issue. Nonetheless, we hope that an improved and extended version of this method to understand breast cancer incidence may contribute to this.

To study the effects of radiation exposure on the transition probabilities in a cell model, a reference group without (significant) radiation exposure is needed to calibrate the model. For this calibration, data from the annual report of the Japanese National Clinical Database from 2018 is used (43). It is presented in tabular form in Table 4.

**Table 4:** Occurrence of breast cancer cases per 100, 000 population in an age group in Japan in 2018, according to Figure 1B in Tada et al. (43).

Age group	Cases	Age group	Cases
20–24	5	60–64	245
25–29	10	65–69	255
30–34	25	70–74	240
35–39	80	75–79	215
40–44	180	80–84	175
45–49	260	85–89	120
50–54	250	90–	60
55–59	230		

Although this data set includes breast cancer incidence among the survivors of the atomic bombs in 1945, this effect is small. It also ignores the difference in breast cancer incidence [Figure 5 in (44)], for example, as a result of differences in lifestyle and medical conditions between the younger and older parts of this population. This is hard to overcome, as such differences will remain for virtually any large calibration group.

Given that our definition of a cancerous cell is very different from a real-world diagnosis resulting in incidence data, a lag period should be considered. This is the period between the existence of the first stable cancerous cell (in our cell model) and an observable tumor. This lag time has been studied in the literature (17), where the values 3.7, 5.1, and 6.1 years were found for different models. All other models (18, 39, 45–47) known by the authors have chosen a fixed lag time of 5 years.

Since the purpose of this study is to demonstrate the feasibility of a computational method and since both the data and our model are too limited to determine this parameter, a lag time of 5 years will be used here, meaning that for the cases reported at an age of *n* years, a cancerous cell is assumed to exist at *n*−5 years.

Assuming that the number of cases follows a Poisson distribution (41), it can be determined what the most likely corresponding rate λ(*a*) as a function of age *a* is for each age group. Ignoring all other aspects, this results in Table 5.

**Table 5:** Assumed Poisson rates in the absence of radiation exposure, where the occurrences from Table 4 have been corrected for a five-year lag period.

Age group	Poisson rate λ(*a*)	Age group	Poisson rate λ(*a*)
15–19	5	55–59	245
20–24	10	60–64	255
25–29	25	65–69	240
30–34	80	70–74	215
35–39	180	75–79	175
40–44	260	80–84	120
45–49	250	85–	60
50–54	230		

To estimate the number of cases after radiation exposure, the *excess relative risk* (R) is used. It modifies the Poisson rate (41) as


$$\begin{array}{l} \tilde{\text{λ}}\left(a,d\right)=\text{λ}\left(a\right)\times \left(1+R\left(a,d\right)\right), \end{array}$$ (5)

where Table 5 is approximately reproduced by the fit


$$\text{λ}(a)=300{\left(\frac{a}{35+a}\right)}^{8}\exp\left[-{\left(a/85\right)}^{6}\right].$$

A very relevant factor in the determination of the excess relative risk (R) is age at menarche (41). The median age at menarche in Japan is 12 years (48). The corresponding curve for a radiation exposure of 1 Gy at age 30 [Figure 2 in (41)] is used to determine the excess relative risk for our radiation exposure scenario with the model assumption that the excess relative risk is linear in *d*.

In this way, the full excess relative risk is given by


$$\begin{array}{l} R\left(a,d\right)=5.2d\frac{25}{a-10}. \end{array}$$

This is all the information needed for a scenario with age at menarche 12 and a radiation exposure of 0.1 Gy at age 30. This leads to the hypothetical number of cases depicted in Table 6. It should be emphasized here that the purpose of the data is to demonstrate the method. The limitations of this study force us to accept shortcomings in the data used, for example, treating all breast cancer cases as ductal carcinoma, when this is evidently not true.

**Table 6:** Assumed Poisson rates for age at menarche 12 years and an exposure to 0.1 Gy at age 30, according to the excess relative risk R from Equation 5.

Age group	Poisson rate $\tilde{\text{λ}}\left(a,0.1\right)$	Age group	Poisson rate $\tilde{\text{λ}}\left(a,0.1\right)$
15–19	5	55–59	343
20–24	10	60–64	319
25–29	25	65–69	288
30–34	98	70–74	250
35–39	269	75–79	206
40–44	337	80–84	158
45–49	360	85–	111
50–54	358		

### 3.2 Fits

Now that we have a cell model, described in Section 2.1 , and an efficient computational model, Section 2.2, to predict cases of breast cancer, and we have Tables 5, 6 with plausible incidence rates, it is possible to look for the parameters *p*_mut_, *p*_die_ and *p*_spr_ that give the best results, where

- *p*_mut_ is the probability that a growth-promoting cell mutation resulting in a stable cell occurs in a time step;
- *p*_die_ is the probability that a growth-stimulating cell mutation resulting in an unstable or hostile cell occurs in a time step;
- *p*_spr_ is the spreading probability that a growth-promoting mutation of a mutated cell spreads to neighboring cells.

These parameters are found using a steepest descent optimization method, with the objective of minimizing the loss function given by the square of the relative differences between the prediction and the number of cases in the table, summed over all age groups. In the experiments performed below, the final value for the loss function will be reported, and although this is hard to interpret as an absolute value, the comparison with the other experiments gives an indication of the fitting success.

Neither the uncertainty in the parameters nor the sensitivity of the predictions with respect to these parameters has been determined, although this is possible to do. The reason for this is that the emphasis here is on the method, its feasibility, and a possible relation between model, data, and optimization method.

The tissue model requires as input a number of cells *N* in the tissue. For the experiments, we have chosen to use *N* = 1, 000, 000 cells. This number of epithelial cells might just represent a subset of the cells present in a female breast. As the cell of origin of breast cancer is not completely known, it remains difficult to pinpoint the number of cells that could undergo radiation-induced carcinogenesis. As absolute numbers of ductal epithelial cells in the human breast are difficult to find in the scientific literature, in contrast to the description of cell types and their relative distribution in the female breast (49) and volumetric analysis of branching (50).

The computation duration of such an experiment is approximately 1 h on a standard office laptop.

#### 3.2.1 Calibration

For the group not exposed to radiation, the same transition probabilities are used at all time steps. For this group, the parameters obtained by optimization are


$$p_{\text{mut}}=0.021,\;p_{\text{die}}=0.495,\;p_{\text{spr}}=0.009,$$

with a loss value of 1.95 × 10^−6^. The corresponding Poisson rates as predicted by the model are given in Table 7. It clearly indicates a qualitative shortcoming of this model. It is not able to explain the decreasing number of breast cancer cases with increasing age. As a result of this, the method estimates the number of cases at ages 35 − 60 much too low.

**Table 7:** Model prediction of the breast cancer incidence rate per age group (per 100, 000) vs. data for the control group without excess exposure to radiation. The prediction was computed using the neighboring cells approximation. The optimal parameter values were found to be *p*_mut_ = 0.020, *p*_die_ = 0.565 and *p*_spr_ = 0.016.

Age group	Data	Prediction	Age group	Data	Prediction
15 − 19	5	4	50 − 54	230	233
20 − 24	10	25	55 − 59	245	240
25 − 29	25	64	60 − 64	255	241
30 − 34	80	112	65 − 69	240	238
35 − 39	180	156	70 − 74	215	230
40 − 44	260	192	75 − 79	175	221
45 − 49	250	217	80 − 84	120	209

This is inconvenient as this phenomenon dominates the outcome of the experiment. This is also true for the experiments below. Due to this fitting problem, the numerical outcomes of the experiment are of limited value. Nonetheless, our main result is that such experiments can be conducted.

#### 3.2.2 Radiation exposure

For the group exposed to radiation, only the parameters *p*_mut_, *p*_die_ and *p*_spr_ are different at time steps around age 30. To be more precise, during 6 time steps (i.e., 6 months), the different set $\left({\tilde{p}}_{\text{mut}},{\tilde{p}}_{\text{die}},{\tilde{p}}_{\text{spr}}\right)$ of transition probabilities is used. For all other time steps, the transition probabilities from the calibration experiment are used.

In this experiment, the parameters during radiation exposure are fitted to yield the best results for the entire data set. The parameters obtained by optimization are


$${\tilde{p}}_{\text{mut}}=0.022,\;{\tilde{p}}_{\text{die}}=0.282,\;{\tilde{p}}_{\text{spr}}=0.008,$$

with a loss value of 8.60 × 10^−6^. The corresponding incidence rates as predicted by the model are given in Table 8.

**Table 8:** Model prediction of the breast cancer incidence rate per age group (per 100, 000) versus data for the control group with excess exposure to radiation at age 30. The prediction was computed using the neighboring cells approximation, assuming an exposure window of 6 months. The optimal parameter values were found to be ${\tilde{p}}_{\text{mut}}=0.022$, ${\tilde{p}}_{\text{die}}=0.289$ and ${\tilde{p}}_{\text{spr}}=0.000$.

Age group	Data	Prediction	Age group	Data	Prediction
15 − 19	5	4	50 − 54	358	238
20 − 24	10	25	55 − 59	343	242
25 − 29	25	64	60 − 64	319	240
30 − 34	98	173	65 − 69	288	234
35 − 39	269	186	70 − 74	250	226
40 − 44	337	206	75 − 79	206	215
45 − 49	360	226	80 − 84	158	204

#### 3.2.3 Exposure duration

The previous experiment can be repeated with a shorter exposure duration of 1 time step (1 month). As the total dose remains constant, this would correspond to a 6 times higher intensity during the period of radiation exposure. The parameters obtained by optimization are


$${\tilde{p}}_{\text{mut}}=0.051,\;{\tilde{p}}_{\text{die}}=0.091,\;{\tilde{p}}_{\text{spr}}=0.000,$$

with a loss value of 8.82 × 10^−6^. The corresponding incidence rates as predicted by the model are given in Table 9.

**Table 9:** Model prediction of the breast cancer incidence rate per age group (per 100, 000) versus data for the control group with excess exposure to radiation at age 30. The prediction was computed using the neighboring cells approximation, assuming an exposure window of 1 month. The optimal parameter values were found to be ${\tilde{p}}_{\text{mut}}=0.051$, ${\tilde{p}}_{\text{die}}=0.091$ and ${\tilde{p}}_{\text{spr}}=0.000$.

Age group	Data	Prediction	Age group	Data	Prediction
15 − 19	5	4	50 − 54	358	237
20 − 24	10	25	55 − 59	343	241
25 − 29	25	64	60 − 64	319	240
30 − 34	98	170	65 − 69	288	235
35 − 39	269	176	70 − 74	250	226
40 − 44	337	204	75 − 79	206	216
45 − 49	360	225	80 − 84	158	204

In this case, the transition probabilities obtained are significantly different. It is not surprising that the mutation probability increases, and the much smaller value of ${\tilde{p}}_{\text{die}}$ might be interpreted as a reduced functionality of the immune system. For the spreading probability, the much smaller value is not straightforward to interpret.

The resulting incidence rates in Table 9 are similar to Table 8. Although this can be an artifact of the fitting method, it corresponds to the idea that the transition probabilities depend on the radiation dose rate, but the breast cancer incidence rate only depends on the total radiation dose.

Although it is interesting to compare these numbers and align them with the phenomena they represent, they should not be given too much significance, as the data and model are not good enough to draw conclusions from. In the end, the main goal of this study is to show that they can be computed.

#### 3.2.4 Number of cells

As the value *N* for the number of epithelial cells in the milk ducts is not known, experiments with a varying number *N* have been performed. The values tested are *N* = 100, 000, *N* = 1, 000, 000 and *N* = 100, 000, 000 cells. Naturally, these numbers lead to slightly different transition probabilities, but the qualitative outcomes are quite similar.

This does not mean that the number of cells is not an important parameter. Above, we have changed the number of cells before fitting to the data, so that the optimizer can adjust somewhat for this. In reality, however, the transition probabilities should not depend (strongly) on the number of cells, so that the number of cells is a relevant parameter. As a result, a larger number of cells would lead to increased incidence rates. As there are no observations demonstrating this phenomenon, this is an interesting topic for further research.

## 4 Discussion

In this study, we have introduced a cell-based numerical approximation method to simulate and study the development of cancerous cells from the epithelial cells of the milk duct. Our methods are much faster than Monte Carlo simulations, which would require computing clusters to perform similar experiments. In this way, the transitions at the cellular level that may lead to cancerous cells can be studied.

The outcomes of our model are not realistic, as a result of oversimplification. The model fails to explain the falling breast cancer incidence rates at higher ages. This complicates the analysis of many other possible simulations as well. A slower rate of cell renewal with increasing age may be a partial explanation for this phenomenon. Additionally, following menopause and with increasing age, regression and atrophy of glandular elements and lobules occur (51). Additionally, a better-suited loss function to optimize may also contribute to this.

Furthermore, considering subpopulations with different sensitivities for radiation-induced cell killing based on cell cycle stage and for radiation-induced mutations based on DNA double-strand break repair pathway availability and genetic status of the cell could refine the model. Additionally, time-dependent transition probabilities could account for variation in sensitivities due to various changing intrinsic and extrinsic factors.

Another improvement could be the implementation of several subpopulations of cells that account for the different radiosensitivities during the cell cycle (G1, S, G2 phase, and mitosis; G0). The percentage of cells in these stages depends, among other things, on tissue type, developmental stage, and menstrual cycle, meaning that this percentage needs to be adapted for these conditions.

A significant difference between this approach and many others is the central role the number of epithelial cells in the milk ducts plays. Although this is not a value that is accessible in surgical practice, it is of crucial importance in this model.

As the aim of this project was to perform an efficient simulation, several assumptions had to be made to reach this. In addition to that, the data limitations have forced us to use a rather restricted set of variables. Both these effects reduce the biological relevance of the simulation results. The authors hope that in future studies, a more complete collection of the relevant data, in combination with a more accurate model, can be used to gain biologically relevant insights.

## Data Availability

The original contributions presented in the study are included in the article/supplementary material, further inquiries can be directed to the corresponding author.

## Footnotes

1 The two-stage clonal expansion model of carcinogenesis encompasses initiation, during which a susceptible stem cell acquires one or more mutations resulting in partially escaped growth control, promotion with clonal expansion of initiated cells, and malignant conversion, during which one of the initiated cells acquires the further genetic changes (16).

2 Evidence accumulates that stem or progenitor cells within the epithelial compartment are the cells of origin in which breast cancer initiation takes place (22). However, transformation under exposure to damaging environmental factors such as ionizing radiation might also occur in differentiated mammary cells (23).

## References

[1] Ron E. Ionizing radiation and cancer risk: evidence from epidemiology. Radiat Res. (1998) 150:S30–41. doi: 10.2307/3579806 9806607

[2] Wade MA Sunter NJ Fordham SE Long A Masic D Russell LJ . c-MYC is a radiosensitive locus in human breast cells. Oncogene. (2015) 34:4985–94. doi: 10.1038/onc.2014.427 25531321 PMC4391966

[3] Cucinotta FA. Non-targeted effects and space radiation risks for astronauts on multiple International Space Station and lunar missions. Life Sci Space Res. (2024) 40:166–75. doi: 10.1016/j.lssr.2023.08.003 38245342

[4] Borak TB Heilbronn LH Townsend LW McBeth RA de Wet W. Quality factors for space radiation: a new approach. Life Sci Space Res. (2014) 1:96–102. doi: 10.1016/j.lssr.2014.02.005 26432594

[5] Cucinotta FA To K Cacao E. Predictions of space radiation fatality risk for exploration missions. Life Sci Space Res. (2017) 13:1–11. doi: 10.1016/j.lssr.2017.01.005 28554504

[6] Cucinotta FA. Biophysics of NASA radiation quality factors. Radiat Prot Dosimetry. (2015) 166:282–9. doi: 10.1093/rpd/ncv144 25883309

[7] Sridharan DM Asaithamby A Bailey SM Costes SV Doetsch PW Dynan WS . Understanding cancer development processes after HZE-particle exposure: roles of ROS, DNA damage repair and inflammation. Radiat Res. (2015) 183:1–26. doi: 10.1667/RR13804.1 25564719

[8] Nguyen QH Pervolarakis N Blake K Ma D Davis RT James N . Profiling human breast epithelial cells using single cell RNA sequencing identifies cell diversity. Nat Commun. (2018) 9:2028. doi: 10.1038/s41467-018-04334-1 29795293 PMC5966421

[9] Liu X Feng D Liu D Wang S Yu X Dai E . Dissecting the origin of breast cancer subtype stem cell and the potential mechanism of malignant transformation. PLoS ONE. (2016) 11:e0165001. doi: 10.1371/journal.pone.0165001 27768723 PMC5074511

[10] Natarajan TG Ganesan N Carter-Nolan P Tucker CA Shields PG Adams-Campbell LL. Gamma-Radiation-induced chromosomal mutagen sensitivity is associated with breast cancer risk in African-American women: caffeine modulates the outcome of mutagen sensitivity assay. Cancer Epidemiol Biomark Prev. (2006) 15:437–42. doi: 10.1158/1055-9965.EPI-05-0353 16537698

[11] Bernstein JL Haile RW Stovall M Boice J John D Shore RE . Radiation exposure, the ATM Gene, and contralateral breast cancer in the women's environmental cancer and radiation epidemiology study. J Natl Cancer Inst. (2010) 102:475–83. doi: 10.1093/jnci/djq055 20305132 PMC2902825

[12] Andrieu N Cavaciuti E Laugé A Ossian K Janin N Hall J . Ataxia-telangiectasia genes and breast cancer risk in a French family study. J Dairy Res. (2005) 72:73–80. doi: 10.1017/S0022029905001147 16180724

[13] Kaufmann WK Filatov L Oglesbee SE Simpson DA Lotano MA McKeen HD . Radiation clastogenesis and cell cycle checkpoint function as functional markers of breast cancer risk. Carcinogenesis. (2006) 27:2519–27. doi: 10.1093/carcin/bgl103 16777992

[14] Poggioli T Sterpone S Palma S Cozzi R Testa A. G0 and G2 chromosomal assays in the evaluation of radiosensitivity in a cohort of Italian breast cancer patients. J Radiat Res. (2010) 51:615–9. doi: 10.1269/jrr.10052 20921829

[15] Bernstein JL Teraoka SN John EM Andrulis IL Knight JA Lapinski R . The CHEK2*1100delC allelic variant and risk of breast cancer: screening results from the Breast Cancer Family Registry. Cancer Epidemiol Biomark Prev. (2006) 15:348–52. doi: 10.1158/1055-9965.EPI-05-0557 16492927

[16] Hazelton WD Clements MS Moolgavkar SH. Multistage carcinogenesis and lung cancer mortality in three cohorts. Cancer Epidemiol Biomark Prev. (2005) 14:1171–81. doi: 10.1158/1055-9965.EPI-04-0756 15894668

[17] Kaiser J Jacob P Meckbach R Cullings H. Breast cancer risk in atomic bomb survivors from multi-model inference with incidence data 1958-1998. Radiat Environ Biophys. (2011) 51:1–14. doi: 10.1007/s00411-011-0387-4 21947564

[18] Eidemüller M Becker J Kaiser J Ulanowski A Apostoaei A Hoffman FO. Concepts of association between cancer and ionising radiation: accounting for specific biological mechanisms. Radiat Environ Biophys. (2023) 62:1–15. doi: 10.1007/s00411-022-01012-1 36633666 PMC9950217

[19] Gray GK Carlson EG Lev T Marshall B Reed AD Sánchez-Covarrubias AP . Defining breast epithelial cell types in the single-cell era. Dev Cell. (2025) 60:2218–36. doi: 10.1016/j.devcel.2025.06.032 40925326 PMC12425455

[20] Lindeman GJ Visvader JE. Insights into the cell of origin in breast cancer and breast cancer stem cells. Asia Pac J Clin Oncol. (2010) 6:89–97. doi: 10.1111/j.1743-7563.2010.01279.x 20565420

[21] MD Anderson UCI UCI and Baylor College of Medicine. The Human Breast Cell Atlas (2022). Available online at: https://navinlabcode.github.io/HumanBreastCellAtlas.github.io/ (Accessed July 12, 2025).

[22] Ingthorsson S Traustadottir GA Gudjonsson T. Cellular plasticity and heterotypic interactions during breast morphogenesis and cancer initiation. Cancers. (2022) 14:5209. doi: 10.3390/cancers14215209 36358627 PMC9654604

[23] Zhang X Powell K Li L. Breast cancer stem cells: biomarkers, identification and isolation methods, regulating mechanisms, cellular origin, and beyond. Cancers. (2020) 12:3765. doi: 10.3390/cancers12123765 33327542 PMC7765014

[24] Carmichael A Sami AS Dixon JM. Breast cancer risk among the survivors of atomic bomb and patients exposed to therapeutic ionising radiation. Eur J Surg Oncol. (2003) 29:475–9. doi: 10.1016/S0748-7983(03)00010-6 12798754

[25] Travis LB Hill DA Dores GM Gospodarowicz M van Leeuwen FE Holowaty E . Breast cancer following radiotherapy and chemotherapy among young women with Hodgkin disease. JAMA. (2003) 290:465–75. doi: 10.1001/jama.290.4.465 12876089

[26] Tokunaga M Land CE Tokuoka S Nishimori I Soda M Akiba S. Incidence of female breast cancer among atomic bomb survivors, 1950-1985. Radiat Res. (1994) 138:209–23. doi: 10.2307/3578591 8183991

[27] Land CE Tokunaga M Koyama K Soda M Preston DL Nishimori I . Incidence of female breast cancer among atomic bomb survivors, Hiroshima and Nagasaki, 1950-1990. Radiat Res. (2003) 160:707–17. doi: 10.1667/RR3082 14640793

[28] Preston DL Ron E Tokuoka S Funamoto S Nishi N Soda M . Solid cancer incidence in atomic bomb survivors: 1958-1998. Radiat Res. (2007) 168:1–64. doi: 10.1667/RR0763.1 17722996

[29] Davis FG Boice JDJ Hrubec Z Monson RR. Cancer mortality in a radiation-exposed cohort of Massachusetts tuberculosis patients. Cancer Res. (1989) 49:6130–6. 2790825

[30] Hada M Georgakilas AG. Formation of clustered DNA damage after high-LET irradiation: a review. J Radiat Res. (2008) 49:203–10. doi: 10.1269/jrr.07123 18413977

[31] Mavragani IV Nikitaki Z Souli MP Aziz A Nowsheen S Aziz K . Complex DNA damage: a route to radiation-induced genomic instability and carcinogenesis. Cancers. (2017) 9:91. doi: 10.3390/cancers9070091 28718816 PMC5532627

[32] Nickoloff JA Sharma N Allen CP Taylor L Allen SJ Jaiswal AS . Roles of homologous recombination in response to ionizing radiation-induced DNA damage. Int J Radiat Biol. (2023) 99:903–14. doi: 10.1080/09553002.2021.1956001 34283012 PMC9629169

[33] Zastko L. Genetic regulation of DNA double-strand breaks and repair pathways. Front Biosci (Scholar Ed). (2025) 17:46225. doi: 10.31083/FBS46225 41504119

[34] Belli C Duso BA Ferraro E Curigliano G. Homologous recombination deficiency in triple negative breast cancer. Breast. (2019) 45:15–21. doi: 10.1016/j.breast.2019.02.007 30818144

[35] Creeden JF Nanavaty NS Einloth KR Gillman CE Stanbery L Hamouda DM . Homologous recombination proficiency in ovarian and breast cancer patients. BMC Cancer. (2021) 21:1154. doi: 10.1186/s12885-021-08863-9 34711195 PMC8555001

[36] Denekamp J. Cell kinetics and radiation biology. Int J Radiat Biol Relat Stud Phys Chem Med. (1986) 49:357–80. doi: 10.1080/09553008514552591 3510997

[37] Weil MM Kittrell FS Yu Y McCarthy M Zabriskie RC Ullrich RL. Radiation induces genomic instability and mammary ductal dysplasia in Atm heterozygous mice. Oncogene. (2001) 20:4409–11. doi: 10.1038/sj.onc.1204589 11466622

[38] Vij V Shpak V Zamotayeva G Lapikura L Ryzhov A Gorokh E . Breast cancer risk in Ukrainian women exposed to Chornobyl fallout while pregnant or lactating: standardized incidence ratio analysis, 1998 to 2016. Eur J Epidemiol. (2022). doi: 10.21203/rs.3.rs-1506950/v1 36197563 PMC10655931

[39] Zupunski L Yaumenenka A Ryzhov A Veyalkin I Drozdovitch V Masiuk S . Breast cancer incidence in the regions of Belarus and Ukraine most contaminated by the Chernobyl accident: 1978 to 2016. Int J Cancer. (2021) 148:1839–49. doi: 10.1002/ijc.33346 33064313 PMC9426215

[40] Goodman MT Cologne JB Moriwaki H Vaeth M Mabuchi K. Risk factors for primary breast cancer in Japan: 8-year follow-up of atomic bomb survivors. Prev Med. (1997) 26:144–53. doi: 10.1006/pmed.1996.9979 9010910

[41] Brenner A Preston D Sakata R Sugiyama H Berrington de González A French B . Incidence of breast cancer in the life span study of atomic bomb survivors: 1958–2009. Radiat Res. (2018) 190:433–44. doi: 10.1667/RR15015.1 30044713 PMC10284068

[42] Cahoon EK. Breast cancer risk among women exposed to fallout after the Chernobyl accident. Int J Epidemiol. (2020) 49:456–8. doi: 10.1093/ije/dyaa038 32215648 PMC7266548

[43] Tada K Kumamaru H Miyata H Asaga S Iijima K Ogo E . Characteristics of female breast cancer in japan: annual report of the National Clinical Database in 2018. Breast Cancer. (2022) 30:157–66. doi: 10.1007/s12282-022-01423-4 36547868 PMC9950166

[44] Kubota K Nakashima K Nakashima K Kataoka M Inoue K Goto M . The Japanese breast cancer society clinical practice guidelines for breast cancer screening and diagnosis, 2022 edition. Breast Cancer. (2023) 31:157–64. doi: 10.1007/s12282-023-01521-x 37973686 PMC10901949

[45] Moolgavkar SH Day NE Stevens RG. Two-stage model for carcinogenesis: epidemiology of breast cancer in females. J Natl Cancer Inst. (1980) 65:559–69. 6931935

[46] Eidemüller M Holmberg E Jacob P Lundell M Karlsson P. Breast cancer risk among Swedish hemangioma patients and possible consequences of radiation-induced genomic instability. Mutat Res. (2009) 669:48–55. doi: 10.1016/j.mrfmmm.2009.04.009 19416732

[47] Heidenreich W Jacob P Paretzke H. Exact solutions of the clonal expansion model and their application to the incidence of solid tumors of atomic bomb survivors. Radiat Environ Biophys. (1997) 36:45–58. doi: 10.1007/s004110050054 9128898

[48] Yoshimi K Matsumura N Takeda T. When and how do adolescent girls in Japan become aware of premenstrual symptoms from menarche? A cross-sectional study among senior high school students. BMJ Open. (2021) 11:e045215. doi: 10.1136/bmjopen-2020-045215 34446479 PMC8395348

[49] Bhat-Nakshatri P Gao H Sheng L McGuire PC Xuei X Wan J . A single-cell atlas of the healthy breast tissues reveals clinically relevant clusters of breast epithelial cells. Cell Rep Med. (2021) 2:100219. doi: 10.1016/j.xcrm.2021.100219 33763657 PMC7974552

[50] Paavolainen O Peurla M Koskinen LM Pohjankukka J Saberi K Tammelin E . Volumetric analysis of the terminal ductal lobular unit architecture and cell phenotypes in the human breast. Cell Rep. (2024) 43:114837. doi: 10.1016/j.celrep.2024.114837 39368089

[51] Russo J Hu YF Silva ID Russo IH. Cancer risk related to mammary gland structure and development. Microsc Res Techn. (2001) 52:204–23.doi: 10.1002/1097-0029(20010115)52:2<204::AID-JEMT1006>3.0.CO;2-F 11169868

